# The Latin American DILI Registry Experience: A Successful Ongoing Collaborative Strategic Initiative

**DOI:** 10.3390/ijms17030313

**Published:** 2016-02-29

**Authors:** Fernando Bessone, Nelia Hernandez, M. Isabel Lucena, Raúl J. Andrade, on behalf of the Latin DILI Network (LATINDILIN) and Spanish DILI Registry

**Affiliations:** 1 Hospital Provincial del Centenario, University of Rosario School of Medicine, Urquiza 3101, 2000 Rosario, Argentina; 2 Hospital de Clínicas, Facultad de Medicina, UdelaR, Av Italia s/n, 11600 Montevideo, Uruguay; hernandez.nelia@gmail.com; 3 Instituto de Investigación Biomédica de Málaga (IBIMA), Hospital Universitario Virgen de la Victoria, Universidad de Málaga, CIBERehd, Blvd. L Pasteur 32, 29071 Málaga, Spain; andrade@uma.es

**Keywords:** hepatotoxicity, drug-induced liver injury, herbals and dietary supplements, registry, Spanish DILI registry, Latin-American DILI registry

## Abstract

Drug induced liver injury (DILI) is a rare but well recognized serious adverse reaction. Pre-marketing studies may not detect liver injury, and DILI becomes very often apparent after the drug is launched to the market. Specific biomarkers for DILI prediction or diagnosis are not available. Toxic liver reactions present with a wide spectrum of phenotypes and severity, and our knowledge on the mechanisms underlying idiosyncratic reactions and individual susceptibility is still limited. To overcome these limitations, country-based registries and multicenter research networks have been created in Europe and North America. Reliable epidemiological data on DILI in Latin America (LA), a region with a large variety of ethnic groups, were however lacking. Fortunately, a LA network of DILI was set up in 2011, with the support of the Spanish DILI Registry from the University of Malaga. The primary aim of the Latin DILI Network (LATINDILIN) Registry was to prospectively identify *bona fide* DILI cases and to collect biological samples to study genetic biomarkers. Physicians involved in the project must complete a structured report form describing the DILI case presentation and follow-up which is submitted to a Coordinator Center in each country, where it is further assessed for completeness. During the last four years, several LA countries (Argentina, Uruguay, Chile, Mexico, Paraguay, Brazil, Ecuador, Peru, Venezuela and Colombia) have joined the network and committed with this project. At that point, to identify both our strengths and weaknesses was a very important issue. In this review, we will describe how the LATINDILI Registry was created. The aims and methods to achieve these objectives will be discussed in depth. Additionally, both the difficulties we have faced and the strategies to solve them will be also pinpointed. Finally, we will report on our preliminary results, and discuss ideas to expand and to keep running this network.

## 1. Introduction

Drug induced liver injury (DILI) is a rare condition characterized by a wide range of phenotypic presentations and several degrees of severity, which lacks specific markers for diagnosis and represents a major concern for patients, physicians, pharmaceutical industry and regulatory bodies. Although any drug incorporated into the market is previously evaluated with respect to its safety profile and tolerability, the number of subjects exposed during clinical evaluation is not sufficient to detect reactions with a frequency of 1 in 10,000 or 1 in 100,000, as it happens with most drugs in terms of DILI. In addition, the study population included in clinical trials is highly selected (some particularly vulnerable subgroups are not considered), and the duration of treatments is not enough to detect late reactions. Indeed, DILI may remain hidden during both the preclinical and clinical phases of drug development. In addition, the number of subjects enrolled in post-marketing studies is usually insufficient to detect the potential for hepatotoxicity from a compound launched into the pharmaceutical market. This is the reason why the actual risk of developing an adverse reaction may only be ascertained when drugs are launched to the market and administered to thousands of patients who may have more than one disease and concomitant intake of other drugs.

As opposed to countries, such as Spain, Iceland, Japan, and the United States [1,2,3,4], where registries and multicenter networks for conducting studies on DILI have existed for several years, Latin America (LA) had not set up this collaborative strategy until only four years ago. Consequently, epidemiological data on DILI in the literature came almost exclusively from the reporting of either isolated cases or small series of subjects [5]. Latin America is a continent comprising 23 countries, showing significant demographic growth in the last few years, and characterized by drug prescription patterns that differ from those in European or North American countries, apart from being associated with high incidence of self-medication [6]. Like in Asia and Africa, throughout Latin America there is a large market for herbs and supplements as an important side of folk medicine, since these products are more accessible and affordable.

Accordingly, we realized the necessity to explore in Latin America the issue of DILI. Hence, we aimed to prospectively identify hepatotoxicity cases induced by drugs and herbals and dietary supplements (HDS) in LA, with detailed information and samples for genetic studies Thus, the Spanish Latin American DILI Registry (SLATINDILI, available online: www.slatindili.uma.es) was created by the end of 2011, after an initiative of and with the ongoing support from the Spanish DILI group.

Adverse drug reactions account for 3.6% to 6.7% of hospital admissions in France and the United States, respectively [7,8]. Spontaneous reporting by the treating physician of adverse reactions during a specific treatment is very useful for early identification of risks factors and for hypothesis generation. However, this methodology is not adequate to learn about the real proportion of drug-induced liver disease cases, but underreporting is possibly its most significant limitation despite obligatoriness. At best, the reporting of hepatotoxicity has been estimated in about 5% of the total number of cases of DILI in a given population [9], probably depending on the experience of the physician facing the adverse reaction and his/her consciousness of the importance of reporting such information. These considerations preclude either quantifying the degree of association, or ensure an adequate and rigorous assessment of causality, and a precise awareness on the population exposed. Lack of sufficient information as to ascertain causality is also seen in the analysis of clinical cases published in medical journals, where the editorial process prior to publication would have to ensure a high level of information demand [10]. An alternative strategy is the creation of a DILI registry, *i.e.*, an information archive system that specifically address DILI cases and that may be structured as a multicenter network centralized in the hospital environment, where the data-collection protocol is shared. Undoubtedly, this always was a very difficult aim in LA both to implement and to keep “alive” this project. It requires not only trained physicians, but especially motivated professionals who recruit the cases and provide detailed information on demographics, description of the adverse reaction, laboratory findings, seriousness, causality, and evolution. The benefits of this kind of initiative are multiple: it increases hepatotoxicity suspicion among physicians, ensures diagnostic certainty by applying a uniform methodology, identifies risk factors, allows formulating working hypotheses, fosters research, and may support regulatory decisions with the consequent impact on public health. DILI registries have provided reliable novel findings on human DILI. In an analysis of 461 patients with DILI identified during the first 10 years of existence of the Spanish DILI Registry [1], 53% required hospitalization, 10% developed chronic DILI, 5% died as a consequence of hepatotoxicity and 2% required a liver transplant.

With the certainty that LA could have the potential to undertake such ambitious project and thanks to both the strong commitment undertaken by the first members of this registry and the design of a clear and factual strategy based on the prior experience in Spain, in 2011 we started working in this network. Today, the Latin DILI Network (LATINDILIN) Registry is a reality. This review describes the steps taken to set up this network, its objectives, preliminary results along with the difficulties overcame and those still pending.

The Latin American DILI registry has the merit of being a network involving different countries, which have some common roots but also great differences regarding ethnicity, prescription patterns and regulatory and drug policies, among others. These differences challenge and stimulate the development of cooperation and team work in order to achieve a common goal.

## 2. What Were Our Goals?

Epidemiological data and disease registries are lacking in LA. Hence, it is not surprising that for DILI, a rare condition, there was no specific registry. The first evaluation of the profile of DILI in LA comes from the analysis of case reports and series published between 1996 and 2012, describing a total of 176 cases (53 drugs) [5]. Ninety percent of the cases came from Chile, Argentina, and Colombia, while Peru, Uruguay, Brazil, Mexico, Venezuela, and Cuba accounted for the remaining 10%. The most frequently reported pharmacological groups were non-steroidal anti-inflammatory drugs (NSAIDs) (32%), anti-infective agents (19%), and anti-androgens (16%), mainly cyproterone acetate. Anti-infective agents predominate in existing DILI registries [1,2,4], while NSAIDs are represented to a lesser extent, and cyproterone acetate is either rare or absent. This over-representation of NSAIDs and the strikingly high incidence of antiandrogenals in LA published case reports may be explained by the publications of case series of nimesulide and cyproterone by Bessone *et al.* [11,12] in Argentina. Another factor that should be considered is that amoxicillin clavulanate combination only has three cases in this review, while DILI registries show that it is the drug that ranks first in the registries [13]. The study by Björnsson *et al.* [2], evaluating the incidence of DILI in Iceland, showed that amoxicillin clavulanate had the highest incidence among all culprit drugs (43 cases per 100,000 exposures).

The retrospective nature of this epidemiological study may explain the over-representation of acute liver failure (ALF) cases in comparison to those found in prospective registries [1,4]. One third of DILI cases considered in this article involved ALF, and this poor outcome was ascribed to 22 drugs (Table 1). It is worth mentioning that no ALF due to acetaminophen overdose was identified among 206 adults with ALF (Villamil F., personal communication). Zapata *et al.* [14] reported on eight cases of paracetamol-induced ALF out of 129 cases collected in two liver transplant centers during a 10-year period. This analysis is the first one to focus on the controversial figures of DILI in our continent, and to provide some epidemiological data on the issue. However, it suffers from the limitations of a methodology based on published cases; which usually tend to be the most serious or those with atypical clinical presentations. Due to these limitations, the low frequency of DILI, and lack of knowledge of the total population exposed to the drug, these studies do not allow to establish incidence data, or to draw reliable conclusions with regard to patient’s demographic and/or phenotypic presentations of DILI.

The sale of over the counter drugs (OTC), *i.e.*, those drugs that, according to regulatory authorities, may be used for the treatment of conditions not requiring the direct supervision of a physician and whose safety is reasonably proved, may have significant impact on the development of hepatotoxicity [15]. An example of this is acetaminophen, a drug that is not only sold over the counter in many countries, but also sold at non-specialized stores. Its safety at therapeutic doses is being questioned, but poisoning (either voluntarily or not) with this drug is a common cause of acute hepatic failure often requiring liver transplantation. On this view, several actions have been taken, such as limiting the number of units in acetaminophen 500 mg packs to 16 tablets for sale at non-specialized stores, and to 32 tablets for sale at pharmacies (UK, 1996) [15]. The impact of this action is difficult to assess, since studies (which are localized and have a short follow-up period) report on different results [16]. The risk of poisoning may also be triggered by the “inadvertent” intake of acetaminophen in drugs which contain it combined with other agents. In 2011, the FDA exhorted manufacturers of prescription drugs with a fixed dose combination containing acetaminophen to limit the amount of acetaminophen to not more than 325 mg per tablet. The FDA also exhorted physicians to refrain from prescribing combinations containing more than such amount, and pharmacists to reject them for sale (March 2014) [17].

The main goal of the creation of the DILI registry in LA was the prospective and standardized identification of the different manifestations that drug-induced liver disease have in this region, so as to obtain highly valuable information regarding subject characteristics, most frequently involved drugs or herbal supplements, phenotypic presentations, and outcomes. Among the main objectives is also the collection of data leading to get a greater understanding on the environmental and host factors in DILI and the comparison with the figures reported by other registries. Examples abound on drugs that have been withdrawn from the market or that have had serious restrictions of use because of hepatotoxicity. The registry information could be very useful to generate risk alerts or to adopt regulatory decisions. A further, more ambitious objective was the creation of a biological sample bank for the study of the genetic markers involved, which would allow to better characterize susceptible individuals, understand the mechanisms of damage, and validate specific biomarkers. This latter possibility would have important implications since, along with the rarity of the condition we do not have specific markers for predicting/diagnosing DILI, with the consequent need for a careful etiological evaluation, which is far from being error-free.

Herbals and dietary supplements-induced liver injury is a growing concern, but the true incidence of HDS-attributable liver injury is unknown. Although 60 different herbal products with liver toxicity have been identified, a thorough causality assessment was lacking in most cases [18,19]. There are several reasons to explain the popularity and increased worldwide use of HDS. Some of them could be associated with the cultural context, but others are related to accessibility, affordability, and mistaken notions of safety, as well as patients’ discontent with conventional medical practice. Herbal remedies and dietary supplements are responsible for nearly 20% of the liver toxicity cases reported in the Drug-Induced Liver Injury Network (DILIN) study (the second most frequent class) [4]. Similarly, complementary and alternative medicines are the second most common etiologies of DILI in China [20]. Two percent of the cases recorded in the Spanish Liver Toxicity Registry are related to herbal remedies [21]. We know that the use of HDS in LA is very common, but there are no data about frequency of use, incidence of liver injury, type of products, similarities and differences with those reported in other countries. Research in herbal liver toxicity represents compelling challenges and it is a priority issue of the SLATINDILI registry.

## 3. How Was the Project Implemented, and What Have We Attained so Far?

Since 1994, the Spanish DILI registry has included 51 centers, and it is based at the *Centro de Investigación Biomédica en Red de Enfermedades Hepáticas y Digestivas (CIBERehd)* (Network Center of Biomedical Research in Hepatic and Digestive Diseases).

Its main goal is the prospective detection and systematic study of suspected DILI in the country, where biological samples for pharmacogenetic are also obtained. With the experience provided by 20 years of continuous work, more than 920 cases have been included so far. The advancement on the understanding in the epidemiology, phenotypic presentation, causality assessment, outcome and underlying mechanisms in DILI provided by this Spanish network has resulted in pioneering, highly-cited contributions to the field of DILI. With the support of this group, and after the identification and collaboration of thought professionals leaders in Argentina, Uruguay, Brazil, Peru, México, Chile, and Venezuela, the registry was set up for idiosyncratic DILI study, and started working as by the end of 2011: the Latin American DILI Network [22,23]. A publication on the methodology and results of the first 300 DILI cases in LA is ongoing, and we would like to present there the novel information of the results in this registry. Meanwhile, we have introduced in the manuscript a statement saying that the reader is referred to the paper by Andrade *et al.*, 2005 [1], which offers a detailed description of the operational structure of the registry, data recording, and case ascertainment in the Spanish DILI Registry. This protocol is also followed at the SLATIN DILI Network.

**Figure 1:**
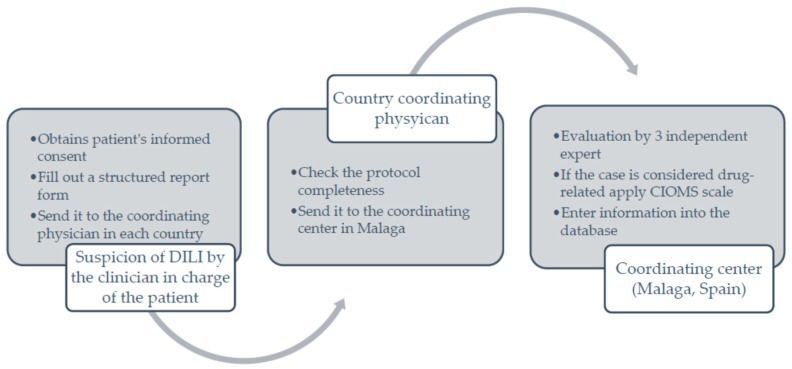
Flow chart depicting the process for case enrollment at the Latin-American drug induced liver injury (DILI) Network.

The *Asociación Latinoamericana para el Estudio del Hígado (ALEH)* (Latin American Association for Liver Study) has supported and accompanied this project since the very beginning, providing the group with a top place in its Website (available online: http://www.alehlatam.org) [24]. The physicians contacted and committed to the project are responsible for spreading it in their respective countries, and for generating—by using the resources most suitable to their local situations—an internal training network for possible DILI cases. Based on the suspicion of DILI, and having the patient’s informed consent following the Spanish model, the treating clinician fills out a standardized form that is sent to the coordinating physician in each country for a first evaluation, and then to the coordinating center in Malaga, Spain. There, the information provided is analyzed, for completeness and the possible association with the drug and drug-drug interactions is evaluated. The causes that were excluded are discussed and, finally, after being evaluated by three independent experts, the event is adjudicated, or not (Figure 1). Hepatitis E virus (HEV) may usually represent a diagnostic problem. HEV determination was carried out in those cases where both clinical context and endemic area have suggested to us a high epidemiological impact of the virus. We are conducting a study analyzing all DILI samples to detect the true incidence of Hepatitis E in this population.

Latin America is considered to have intermediate prevalence rates of Hepatitis A virus (HAV) [25]. Data about HEV prevalence is scarce, and displays significant regional variations. Many Latin American countries have reported patients with acute hepatitis E by anti-HEV IgM and/or HEV RNA detection, and even if LA is often considered highly endemic for this virus, there is no evidence to support it [26]. Data published in 1997 from Uruguay, where 214 patients were studied showed that 2.8% were anti HEV positive (EIA, Abbot). Moreover, a sample of 252 blood donors from the National Blood Service was analyzed, and five persons were found to be anti HEV positive, with only 3 (1.2%) being confirmed in the Center for Disease Control in the United States [27].

Neither HAV nor HEV infection are considered between the most important causes of acute liver failure in Latin America. Mendizabal *et al.* [28], from Argentina, carried out a retrospective analysis of 154 adult patients with ALF, who were admitted in six liver transplantation programs (from June 2005 to December 2011). The most frequent causes of ALF were: viral hepatitis B (30%), autoimmune hepatitis (26%), and indeterminate causes (26%). Only two had acute HAV, and 1 had herpes simplex virus. No cases of ALF due to hepatitis C, D, or E were identified. No acetaminophen (ACM) overdose linked to liver toxicity was reported so far in our LA registry [28].

An analysis of our database, from a total of 51 excluded cases, the causes of exclusion were as follow: do not fulfill the criteria for DILI (*n* = 8); Viral serology is required to rule out viral hepatitis in this episode (*n* = 16); no information available about start and stop days of treatment (*n* = 15); imaging test not available for the DILI episode (*n* = 1); hepatic disease with other etiology: autoimmune hepatitis (*n* = 4), biliary obstruction (*n* = 1), ischemic hepatitis (*n* = 1), hepatitis A (*n* = 1), hepatitis C reactivation (*n* = 1), hepatitis E (*n* = 2); alcoholic hepatitis (*n* = 1).

Once the liver damage is considered to be drug-related, the Council for International Organizations of Medical Sciences (CIOMS)/the Roussel Uclaf Causality Assessment Method (RUCAM) scale is applied to assign the score for probability of the suspicion, and all the information is entered into a database. Usually, DILI cases are intended to be followed up to resolution.

We used the RUCAM scale version as discussed at the International Consensus meeting and published by Danan *et al.* in 1993 [29]. Causality grading was analyzed in 197 cases, and the results were as follow: highly probable 17 (9%); probable 133 (67%) and possible 47 (24%). Ethical requirement for the safeguarding of the privacy and confidentiality of personal data have been implemented.

Since it started working almost four years ago, the group has maintained communication through periodic teleconferences and working meetings, usually during the congresses held on this specialty. With the aim of communicating its existence and stimulating the incorporation of more professionals, several abstracts have been presented in international and local meetings and Conferences, and discussion forums have been organized with clinical cases and oral presentations on DILI. In addition, several collaborative articles were published, providing significant contributions to the understanding of DILI [30,31,32]. The information from the participating centers, the minutes of the meetings held, and the scientific production can all be checked in the Web page (available online: www.slatindili.uma.es) especially created for that purpose. Thus, not only have the working groups existing at the beginning been enlarged, but also professionals from other countries, such as Paraguay, Ecuador, and Colombia have joined, bringing us closer to the aim of representing each and every country in the region.

## 4. Preliminary Results from Our Registry

By following the methodology described above, more than 250 well-vetted DILI cases from Argentina, Uruguay, Chile, Mexico, Paraguay, Venezuela, Ecuador, Brazil, and Peru have been registered so far. The average age was 51 years (15–89 years), and the female gender was the predominant one (59%). When a sample of 206 patients was analyzed, we observed that most of DILI cases came from Argentina (96; 47%); followed by Uruguay (66; 32%), Chile (15; 7%); Mexico (10; 5%), Paraguay (7; 3%), Venezuela (6; 3%); Ecuador (4; 2%); Brazil (1; 0.5%), and Peru (1; 0.5%).

Hepatocellular presentation was observed only in 54% of the cases. Anti-infectives, musculoskeletal agents, and sex hormones were the most commonly involved therapeutic groups. The main 10 drugs involved and leading the LA DILI Registry were as follow: Amoxicillin-clavulanate (*n* = 20), diclofenac (*n* = 13), nimesulide (*n* = 11), nitrofurantoin (*n* = 11), cyproterone acetate (*n* = 9), ibuprofen (*n* = 7), RIP + INH + PIZ (*n* = 7), carbamazepine (*n* = 5), phenytoin (*n* = 4) and thiamazole (*n* = 4). Amoxicillin clavulanate also ranks first in the Spanish and American registries (Table 2). The comparison of demographics and clinical parameters with the data obtained from the cases included in the Spanish DILI Registry (Table 3) showed patients had younger age, female gender predominance and a lower rate of hepatocellular injury at presentation in the Latin American registry.

Although both LATIN and Spanish registries of DILI have amoxicillin-clavulanate as the main causative agent, in the Spanish network this antibacterial combination is followed by tuberculostatics, ibuprofen, and atorvastatin while, in the LATINDILIN, it is followed by diclofenac, nimesulide and nitrofurantoin. The differences between both registries could be tentatively explained by geographical variations or differences in patterns of drug use [33].

The most recent results published from the DILIN (*n* = 899) show similar patterns of DILI presentation to what has been observed in LA: average age of 49 years, 59% of female gender, jaundice in 70% of the cases, and hepatocellular presentation in 54% of them [4].

Regarding HDS, 22 cases (10%) have been registered. Seven of them were adjudicated to a dietary supplement for body building (mainly stanozolol) and 15 cases to herbs, including Herbalife^®^ products (*n* = 3), Garcinia cambogia (*n* = 2), Lipodex (*n* = 2), Ruta graveolans, Centella Asiatica, Chitosan^®^/Acacia rigidula, Echinacea, Equisetum arvense, Ginkgo biloba, Hydroxycut^®^, and Monascus purpureus.

Recent genome wide association (GWA) studies have revealed a number of single nucleotide polymorphisms in the human leukocyte antigen (*HLA*) region associated with idiosyncratic hepatotoxicity attributed to specific drugs (amoxicillin-clavulanate, flucloxacillin, lumiracoxib, lapatinib) [34,35,36,37]. These extremely promising findings were made possible thanks to the existence of registries and working networks that identified and centralized drug-induced hepatotoxicity cases. They are yet another example of the fact that registries greatly contribute to improving our knowledge on DILI mechanism, and the results of their work will probably guide diagnostic methodologies in the future to move toward a precision medicine.

We collected blood samples at DILI recognition for both GWA Studies and drug-polymorphism analysis, but we intend to participate in the recently setup Pro Euro DILI Registry [38]. During the period the LA Registry has been operating, 85 blood samples have been collected, several of which have already been included in GWA studies; this proves that LA—as one of the proposals with the largest scope in our project—has already started being represented in pharmacogenomics studies.

The fact that DILI cases in GWA studies were enriched in amoxicillin-clavulanate and flucoxacillin cases have made detection of signals unrelated to these compounds difficult. Hence, a GWA studies involving new and previous DILI cases excluding cases relating to flucloxacillin and co-amoxiclavulanic was recently performed. This study reported a novel genome-wide significant association of HLA-A*33:01 with all causes of DILI. The most important conclusion from the authors is that the novel genome-wide significant association of HLA-A*33:01 with DILI as a whole, together with its strong association with DILI from both terbinafine and other unrelated compounds, confirms an important role for adaptive immunity in the pathogenesis of the disease, and widens the range of drugs associated with idiosyncratic DILI for which there is a *HLA* risk factor [39].

## 5. Where Do We Stand Today and What Are We Planning for the Future?

The LATINDILI Registry is still a young network but has managed to consolidate itself as a working group, and is positioning in the specialized international community due to its high scientific quality. The epidemiological data being collected from the identification and the methodical prospective registration of DILI cases in our region represent not only the attainment of one of our objectives, but also the main incentive to continue with this ambitious project. It is essential to continue growing, and to do so at a steady pace, by potentiating the contribution of the centers already incorporated, the incorporation of more countries so as to attain the representativeness required for a more extensive analysis, and the sustained publication of results. As we see it, increasing communication of the Registry existence, as well as its work and results, is an essential mean to encourage incorporation. The proposals to reach this goal include (1) the organization of conferences showing the project at various universities and scientific associations in LA; (2) the identification of more specialized professionals who, in addition to actively participate, can act as communicators; (3) the getting off a place in local Web pages; and (4) the expansion and update of the information in the Web pages already available. Much of this work may be facilitated and conducted by ALEH, with which we count on to communicate, establish close links, and make the required contacts.

In addition, with the unconditional support of the Spanish DILI group, actions are being taken to develop and implement strategies aimed to the formation and promotion of scientific research, in order to generate knowledge based on principles of excellence and cooperation.

Among medium-term objectives, there is the implementation of training courses focused on the study of the mechanisms underlying DILI and provide a framework for cooperation and information exchange of scientific findings and favoring short term scientific missions. This collaborative and interdisciplinary network will aim to (1) establish the characteristics of DILI expression, search for risk factors and evaluate the outcome; (2) improve the instruments for causality assessment; (3) increase knowledge on etiopathogenic mechanisms and identification of susceptible subjects; and (4) help develop diagnostic and predictive biomarkers in DILI.

## 6. Summary and Conclusions

The diagnosis of DILI requires physicians’ awareness of the condition to generate suspicion, along with clinical skills to critically evaluate the heterogeneous phenotypes of liver toxicity.

We have presented here the main objectives, and how was our LA project implemented. In addition, we also described our main achievements and future directions.

The strong commitment of countries included in the LATINDILIN progressively contributed to enlarge the number of patients over time.

More than 250 patients enrolled throughout LA during the last five years is clear evidence that our DILI registry is successfully working up to now.

In the long run, this project should provide the information required to facilitate the adoption of regulatory measures, and should also be an instrument for public health protection. Prospective registries in DILI have proved to be the best tool to increase our understanding of this complex and intriguing disorder, the identification of population at risk and to foster the implementation of a safety personalized medicine.

## Figures and Tables

**Table 1:** Main therapeutic classes and individual agents with progression to ALF (acute liver failure) or OLT (orthotopic liver transplantation) published in Latin America (LA) during 1996–2012 [5].

Therapeutic Class	*n* (%)	ALF/OLT *n* (%)	Individual Agent
NSAIDs and anti-rheumatic drugs	62 (32)	16 (26)	Nimesulide, Piroxicam, Diclofenac, Naproxen
Anti-infectious	37 (19)	11 (30)	Nitrofurantoin, Isoniazid, Trovofloxacin, Clarithromycin
Genito-urinary system and sex hormones	34 (18)	14 (22)	Progesterone, Cyproterone Acetate, Flutamide
Antineoplastic and immunomodulators	10 (5)	2 (3)	Imatinib, Tamoxifen
Anticonvulsants	7 (4)	3	Valproic Acid, Phenytoin
Cardiovascular system	6 (3)	1	Methyldopa
Anti-virals	5 (3)	1	Nevirapine
Anesthetics	5 (3)	5	Halothane
Antithyroid	5 (3)	4	Propylthiouracil
Other groups	20	5	Ketoconazole, Griseofulvin, Disulfiram, Mycophenolate

**Table 2:** Top ten individual agents that cause DILI in the LA Registry. Comparisons among Spanish drug induced liver injury (DILI) Registry [33] and drug induced liver injury network (DILIN) study [4].

Individual Agent (*n*)	Latin DILI Network (LATINDILIN) Registry (*n* 206)	Spanish DILI Registry (*n* 867) *	DILIN Study (*n* 899)
Amoxicillin-clavulanate	20	186	91
Diclofenac	13	16	12
Nimesulide	11	9	-
Nitrofurantoin	11	-	42
Cyproterone acetate	9	3	-
Ibuprofen	7	22	1
RIP + INH + PIZ#	7	29	-
Carbamazepine	5	8	4
Phenytoin	4	3	12
Thiamazole	4	7	3

* Only cases with a single culprit drug; # RIP: rifampicin, INH: isoniazid, PIZ: pyrazinamide. Forty-eight cases were adjudicated to INH in the DILIN network.

**Table 3:** Comparative data between the Spanish and the LATINDILI Registries [33].

Variables	LATINDILI Registry (200)	Spanish DILI Registry (867)	*p* Value
Mean age (years)	51	54	0.02
Female (%)	59	49	0.01
Jaundice (%)	67	68	NS
Fatal cases (%)	4.6	4	NS
Hepatocellular damage (%)	54	63	0.03

NS: non-significant *p* value.

## Abbreviations

DILI	Drug induced liver injury
LA	Latin America
ALEH	Latin American Association for Liver Study
CIOMS	Council for International Organizations of Medical Scientists
RUCAM	Roussel Uclaf Causality Assessment Method
NSAIDs	non-steroidal anti-inflammatory drugs
GWA	genome wide association
HDS	herbals and dietary supplements
